# Supplementary material for: HIVprotI: an integrated web based platform for prediction and design of HIV proteins inhibitors
Source: J Cheminform. 2018 Mar 9;10:12. doi: 10.1186/s13321-018-0266-y (PMC5845081; doi:10.1186/s13321-018-0266-y)
Supplement: Supplementary file 2 — Additional file 2. Source code of HIVProtI web server. [file 13321_2018_266_MOESM2_ESM.zip › HIVprotI_Source-code/hivprotI/weka-3-6-6/doc/weka/attributeSelection/package-summary.html]

weka.attributeSelection


---


|  |  |  |
| --- | --- | --- |
| **Overview** \| **Package** \| Class \| **Tree** \| **Deprecated** \| **Index** \| **Help** \| **Weka's home** | |  |
| **PREV PACKAGE**   **NEXT PACKAGE** | **FRAMES**    **NO FRAMES**     **All Classes** |  |


---

## Package weka.attributeSelection

| **Interface Summary** | |
| --- | --- |
| **AttributeEvaluator** | Interface for classes that evaluate attributes individually. |
| **AttributeTransformer** | Abstract attribute transformer. |
| **ErrorBasedMeritEvaluator** | Interface for evaluators that calculate the "merit" of attributes/subsets as the error of a learning scheme |
| **RankedOutputSearch** | Interface for search methods capable of producing a ranked list of attributes. |
| **StartSetHandler** | Interface for search methods capable of doing something sensible given a starting set of attributes. |
| **SubsetEvaluator** | Interface for attribute subset evaluators. |

| **Class Summary** | |
| --- | --- |
| **ASEvaluation** | Abstract attribute selection evaluation class |
| **ASSearch** | Abstract attribute selection search class. |
| **AttributeSelection** | Attribute selection class. |
| **AttributeSetEvaluator** | Abstract attribute set evaluator. |
| **BestFirst** | BestFirst:    Searches the space of attribute subsets by greedy hillclimbing augmented with a backtracking facility. |
| **CfsSubsetEval** | CfsSubsetEval :    Evaluates the worth of a subset of attributes by considering the individual predictive ability of each feature along with the degree of redundancy between them.    Subsets of features that are highly correlated with the class while having low intercorrelation are preferred.    For more information see:    M. |
| **CheckAttributeSelection** | Class for examining the capabilities and finding problems with attribute selection schemes. |
| **ChiSquaredAttributeEval** | ChiSquaredAttributeEval :    Evaluates the worth of an attribute by computing the value of the chi-squared statistic with respect to the class.    Valid options are: |
| **ClassifierSubsetEval** | Classifier subset evaluator:    Evaluates attribute subsets on training data or a seperate hold out testing set. |
| **ConsistencySubsetEval** | ConsistencySubsetEval :    Evaluates the worth of a subset of attributes by the level of consistency in the class values when the training instances are projected onto the subset of attributes. |
| **CostSensitiveASEvaluation** | Abstract base class for cost-sensitive subset and attribute evaluators. |
| **CostSensitiveAttributeEval** | A meta subset evaluator that makes its base subset evaluator cost-sensitive. |
| **CostSensitiveSubsetEval** | A meta subset evaluator that makes its base subset evaluator cost-sensitive. |
| **ExhaustiveSearch** | ExhaustiveSearch :     Performs an exhaustive search through the space of attribute subsets starting from the empty set of attrubutes. |
| **FilteredAttributeEval** | Class for running an arbitrary attribute evaluator on data that has been passed through an arbitrary filter (note: filters that alter the order or number of attributes are not allowed). |
| **FilteredSubsetEval** | Class for running an arbitrary subset evaluator on data that has been passed through an arbitrary filter (note: filters that alter the order or number of attributes are not allowed). |
| **GainRatioAttributeEval** | GainRatioAttributeEval :    Evaluates the worth of an attribute by measuring the gain ratio with respect to the class.    GainR(Class, Attribute) = (H(Class) - H(Class \| Attribute)) / H(Attribute).    Valid options are: |
| **GeneticSearch** | GeneticSearch:    Performs a search using the simple genetic algorithm described in Goldberg (1989).    For more information see:    David E. |
| **GreedyStepwise** | GreedyStepwise :    Performs a greedy forward or backward search through the space of attribute subsets. |
| **HoldOutSubsetEvaluator** | Abstract attribute subset evaluator capable of evaluating subsets with respect to a data set that is distinct from that used to initialize/ train the subset evaluator. |
| **InfoGainAttributeEval** | InfoGainAttributeEval :    Evaluates the worth of an attribute by measuring the information gain with respect to the class.    InfoGain(Class,Attribute) = H(Class) - H(Class \| Attribute).    Valid options are: |
| **LatentSemanticAnalysis** | Performs latent semantic analysis and transformation of the data. |
| **LFSMethods** |  |
| **LinearForwardSelection** | LinearForwardSelection:    Extension of BestFirst. |
| **OneRAttributeEval** | OneRAttributeEval :    Evaluates the worth of an attribute by using the OneR classifier.    Valid options are: |
| **PrincipalComponents** | Performs a principal components analysis and transformation of the data. |
| **RaceSearch** | Races the cross validation error of competing attribute subsets. |
| **RandomSearch** | RandomSearch :     Performs a Random search in the space of attribute subsets. |
| **Ranker** | Ranker :     Ranks attributes by their individual evaluations. |
| **RankSearch** | RankSearch :     Uses an attribute/subset evaluator to rank all attributes. |
| **ReliefFAttributeEval** | ReliefFAttributeEval :    Evaluates the worth of an attribute by repeatedly sampling an instance and considering the value of the given attribute for the nearest instance of the same and different class. |
| **ScatterSearchV1** | Class for performing the Sequential Scatter Search. |
| **SubsetSizeForwardSelection** | SubsetSizeForwardSelection:    Extension of LinearForwardSelection. |
| **SVMAttributeEval** | SVMAttributeEval :    Evaluates the worth of an attribute by using an SVM classifier. |
| **SymmetricalUncertAttributeEval** | SymmetricalUncertAttributeEval :    Evaluates the worth of an attribute by measuring the symmetrical uncertainty with respect to the class. |
| **UnsupervisedAttributeEvaluator** | Abstract unsupervised attribute evaluator. |
| **UnsupervisedSubsetEvaluator** | Abstract unsupervised attribute subset evaluator. |
| **WrapperSubsetEval** | WrapperSubsetEval:    Evaluates attribute sets by using a learning scheme. |

---


|  |  |  |
| --- | --- | --- |
| **Overview** \| **Package** \| Class \| **Tree** \| **Deprecated** \| **Index** \| **Help** \| **Weka's home** | |  |
| **PREV PACKAGE**   **NEXT PACKAGE** | **FRAMES**    **NO FRAMES**     **All Classes** |  |


---
